# Takayasu arteritis detected by PET/MRI with ^18^F-fluorodeoxyglucose

**DOI:** 10.1007/s12350-018-1431-4

**Published:** 2018-09-20

**Authors:** Yasuchika Takeishi, Takatoyo Kiko, Tetsuro Yokokawa, Hiroyuki Kunii, Shohei Ichimura, Shiori Takizawa, Atsuro Masuda, Takashi Kaneshiro, Takuya Ando, Seiichi Takenoshita

**Affiliations:** 1 Department of Cardiovascular Medicine, Fukushima Medical University, 1 Hikarigaoka, Fukushima, 960-1295 Japan; 2 Advanced Clinical Research Center, Fukushima Medical University, Fukushima, Japan

## Introduction

Takayasu arteritis is an auto-immune inflammatory disease, which involves the aorta and its major branches, and is common in young to middle-aged women. The inflammation results in narrowing of arteries, and coronary arteries are sometimes involved in Takayasu arteritis, leading to lethal events.^1^ It has been reported that PET/CT with ^18^F-fluorodeoxyglucose (FDG) is sensitive to detect recurrence in Takayasu arteritis.^2^,^3^ Here, we present ^18^F-FDG PET/ MRI images of Takayasu arteritis with coronary stenoses.

## Case Summary

**Figure 1:**
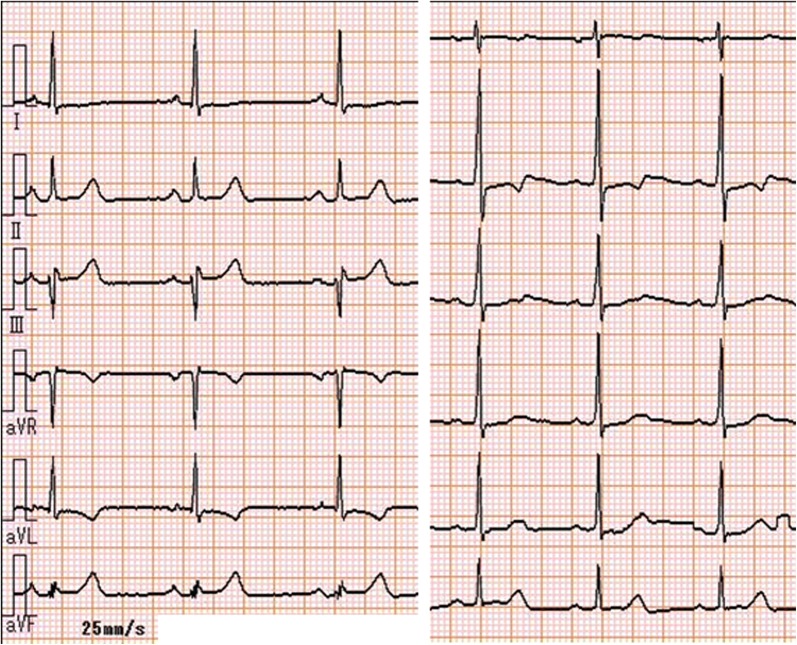
Electrocardiogram on admission. ST-segment depression was observed in aVL, V_2_ to V_5_ leads

**Figure 2:**
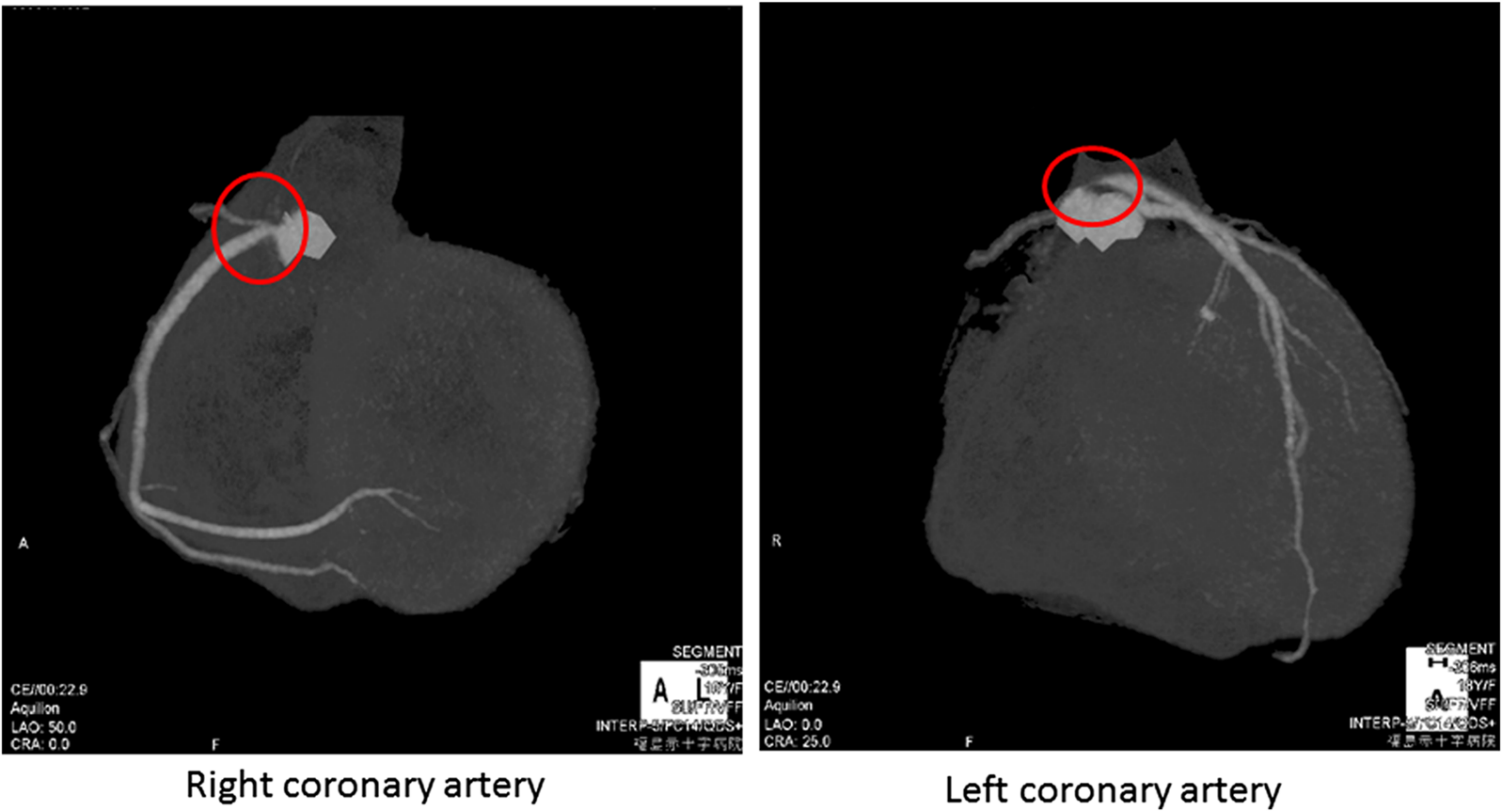
Coronary CT angiography. Severe stenoses were observed in the ostium of the left main trunk and the right coronary artery

**Figure 3:**
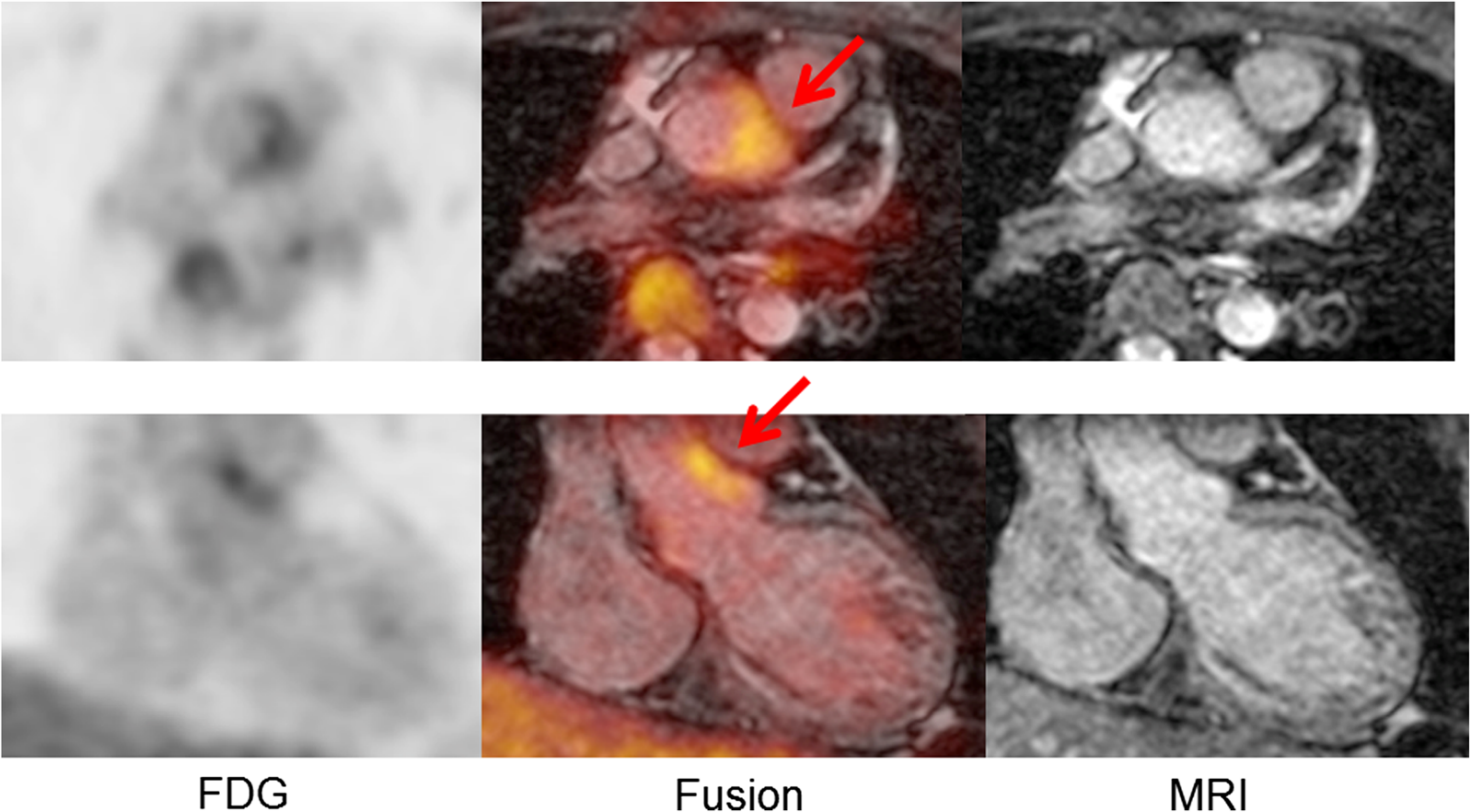
^18^F-FDG PET/MRI images of a patient with Takayasu arteritis. Arrows indicate FDG uptakes on the aortic root, suggesting active vasculitis in the aortic wall and coronary ostial lesions

A 15-year-old woman presented with a chief complaint of chest compression on effort. She did not have coronary risk factors, infectious diseases, or congenital heart diseases. Her electrocardiogram showed ST-segment depression in precordial leads (Figure 1). Two-dimensional echocardiography revealed normal left ventricular function and mild to moderate aortic regurgitation. Contrast-enhanced CT showed neither stenosis nor specific abnormalities in the aorta and its main branches. However, since ostial stenoses of the right and left coronary arteries were suspected by coronary CT angiography (Figure 2), we performed invasive coronary angiography and found 99% stenoses in the ostium of the left main trunk and the right coronary artery. She was started on medical treatment, including beta-blocker, antiplatelet, and statin. Her C-reactive protein, serum amyloid A, and erythrocyte sedimentation rate were high, and systemic inflammation was suggested. She was diagnosed as Takayasu arteritis, and oral administration of prednisolone was started. ^18^F-FDG PET/MRI revealed an intense uptake of FDG in the aortic root (Figure 3). Follow-up coronary angiography demonstrated slight regression of coronary ostial stenosis after immunosuppressive therapy. *She had no angina and ischemic ST-segment change after 6*-*minute ergometry. Percutaneous coronary intervention with coronary stent has high risk of restenosis, and coronary artery bypass surgery was considered after suppression of its disease activity*. In this rare case of Takayasu arteritis with limited inflammatory lesions in the aortic root and coronary ostium, ^18^F-FDG PET/MRI provided useful anatomical information for the localization of vasculitis.
